# Optimizing Imaging Adherence in Acute Pancreatitis: A Clinical Audit at a District General Hospital

**DOI:** 10.7759/cureus.94967

**Published:** 2025-10-20

**Authors:** Shreya Pal, Matthew Oxenham, Manoj Chand, Jay Allu

**Affiliations:** 1 Department of General Surgery, East Kent Hospitals University NHS Foundation Trust, Ashford, GBR

**Keywords:** acute pancreatitis, clinical audit, clinical guidelines, ct scan, imaging

## Abstract

Background: In patients presenting with symptoms suggestive of acute pancreatitis, routine imaging is not necessary when abdominal pain and elevated serum amylase or lipase are present without signs of severe disease. Early CT scans may not accurately reflect disease severity and provide limited diagnostic benefit.

Materials and methods: A retrospective clinical audit was conducted at a district general hospital in the United Kingdom to evaluate current imaging practices in acute pancreatitis. Adult patients presenting to the accident and emergency (A&E) department with symptoms of acute pancreatitis were included. Two audit cycles were performed: January-March 2023 (Cycle 1) and April-May 2024 (Cycle 2). Imaging timing, modality, and adherence to standards recommended by the Royal College of Radiologists were assessed. Interventions to improve guideline compliance were implemented between cycles.

Results: In Cycle 1, 44 patients presented with acute pancreatitis symptoms; 24 (54%) underwent CT scans, of which 15 were performed within 72 hours. Serum amylase was checked in all patients. Imaging findings consistent with pancreatitis were observed in 80% of cases, including one case of pancreatic necrosis, seven cases of intraperitoneal fluid, and five cases of peripancreatic fat stranding. Ultrasound within 24 hours was infrequently performed. Post-intervention (Cycle 2), 32 patients were assessed, with 15 (46.8%) undergoing CT scans, of which three were performed within 72 hours. Serum amylase was checked in all patients. Awareness efforts, including departmental presentations and posters, contributed to improved compliance.

Conclusions: This closed-loop clinical audit demonstrates that targeted interventions and increased awareness of imaging guidelines can improve adherence among clinicians, reducing unnecessary radiation exposure and optimizing CT scan utilization in acute pancreatitis.

## Introduction

Acute pancreatitis is an acute inflammatory condition of the pancreas presenting with abdominal pain and elevated pancreatic enzymes, with an incidence of approximately 13-45 per 100,000 annually worldwide [1]. The initial diagnosis is typically clinical, supported by biochemical markers such as raised serum amylase or lipase. Imaging plays a crucial role in confirming diagnosis, assessing severity, and identifying complications, but indiscriminate use, especially of early computed tomography (CT), may be unnecessary and potentially harmful.

Royal College of Radiologists (RCR) standards recommend avoiding routine early CT (within 72 hours) in uncomplicated acute pancreatitis due to limited sensitivity for detecting pancreatic necrosis early in the disease course and lack of impact on immediate management [2]. Early ultrasound within 24 hours is advised to evaluate for biliary etiology, a common cause of acute pancreatitis [3]. Overuse of CT exposes patients to ionizing radiation, which carries cumulative risks, as highlighted in UK government safety advice [4,5].

One study found that more than half of patients with acute pancreatitis underwent unnecessary CT imaging in the emergency department, despite diagnosis already being established by non-imaging diagnostic criteria. This imaging did not improve patient outcomes, as the hospital stay duration did not change compared to patients who did not have an early CT scan. However, it generated nearly $1 million in excess costs, highlighting a substantial opportunity for cost savings by reducing avoidable imaging [6]. For patients, unnecessary CT scans increase radiation exposure, which is a significant clinical concern. In 2023, an estimated 93 million CT examinations in the United States were projected to result in approximately 103,000 future radiation-induced cancers, with the most significant burden arising from abdomen, pelvis, and chest imaging. Although cancer risks were proportionally higher in children, adults accounted for most cases due to higher CT utilization. If current practices persist, CT-associated cancers could represent up to 5% of new cancer diagnoses annually [7].

This clinical audit evaluates current imaging practices for acute pancreatitis at a district general hospital (DGH). It implements interventions aimed at improving adherence to RCR standards, thereby optimizing patient care and resource utilization.

## Materials and methods

Setting

This audit was conducted at a district general hospital that provides acute and elective services for a population of approximately 700,000 people. The hospital trust provides both emergency and routine care and is the main point of care for patients presenting with suspected acute pancreatitis. It is, hence, an appropriate setting for reviewing current imaging practice and adherence to recommended standards, given the clinical significance of this condition.

Audit standards

The audit was based on the RCR guidelines for imaging in acute pancreatitis [2]. According to these guidelines, an initial CT scan should ideally be performed 72-96 hours after the onset of symptoms, unless earlier imaging is required due to clinical concern. Evidence demonstrates that CT performed very early in the disease course may not accurately demonstrate the extent of severity, particularly pancreatic necrosis, and therefore may provide limited diagnostic and prognostic benefit [8,9].

CT imaging is specifically indicated when there is diagnostic uncertainty, when severe pancreatitis is suspected, or when the patient’s clinical condition deteriorates. This targeted approach helps ensure appropriate use of imaging while avoiding unnecessary radiation exposure and contrast administration in patients who do not require it [10].

In addition to CT, the guidelines recommend that patients undergo an ultrasound of the gallbladder within 24 hours of diagnosis. This is an important step in establishing gallstone disease as a potential underlying cause of acute pancreatitis and guiding subsequent management decisions such as cholecystectomy or ERCP [10,11].

When CT imaging is undertaken, reports require documenting the presence or absence of key features of severity, namely pancreatic necrosis, free intraperitoneal fluid, and peripancreatic fat stranding. Structured reporting of these findings supports accurate assessment of disease severity and informs multidisciplinary management planning [9]. The RCR advised that the target compliance rate for all standards be set at 95%.

Design

The audit was designed as a retrospective clinical audit and was conducted in two cycles to allow for re-evaluation after an intervention. The first cycle covered the period from January to March 2023, while the second cycle reviewed cases between April and May 2024. This two-cycle approach enabled the team to identify baseline performance, implement targeted interventions, and then assess whether improvements had been achieved.

Population

The audit population consisted of adult patients who presented to the accident and emergency (A&E) department with symptoms suggestive of acute pancreatitis. These cases were identified through the hospital’s clinical audit department, ensuring that the case selection was systematic and unbiased.

Data collection

Data were studied from electronic medical records and radiology databases. Information collected included patient demographics, serum amylase levels, and details of imaging performed, including CT, ultrasound, and magnetic resonance cholangiopancreatography. The timing of imaging was recorded relative to both symptom onset and hospital admission, as this is central to assessing compliance with RCR guidance.

For CT scans, reports were examined for documentation of pancreatic necrosis, free fluid, and peripancreatic fat stranding. This ensured not only that imaging was performed appropriately but also that reports contained the necessary information for guiding clinical management. In addition, clinical indications for imaging and radiologist comments regarding timing were reviewed. Collecting this information allowed the audit to determine whether imaging was justified and whether radiological practice was aligned with the recommended standards.

The CT scans included in this study were performed across multiple scanners (at least eight, including mobile units) within the trust, with varied CT settings. All scans were CT abdomen and pelvis with portovenous phase contrast. While individual scan details are not reported due to the large number of images, these variations reflect routine clinical practice in our setting.

Intervention

Following completion of the first cycle of the audit, the findings were presented at departmental meetings, ensuring that clinicians were aware of current practice in relation to standards. To reinforce key messages, educational posters summarizing the guidelines were displayed in the A&E department and the General Surgery department, targeting the clinical teams most directly involved in the early management of acute pancreatitis. Furthermore, discussions were held with radiology staff regarding the importance of structured CT reporting, as well as the guideline-based timing of imaging [12].

These interventions were designed to address both knowledge gaps and practical barriers, with the ultimate goal of improving patient care and ensuring patients with acute pancreatitis receive timely, appropriate, and standardized imaging and management.

## Results

Patient demographics

Table 1 shows the patient demographics for the first and second cycles of the audit.

**Table 1:** Number of patients presenting with symptoms of acute pancreatitis to the emergency department

Audit cycle	Total number of patients	Median age	Male-to-female ratio
Cycle 1	44	68	1:1.15
Cycle 2	32	57	1:0.6

**Figure 1:**
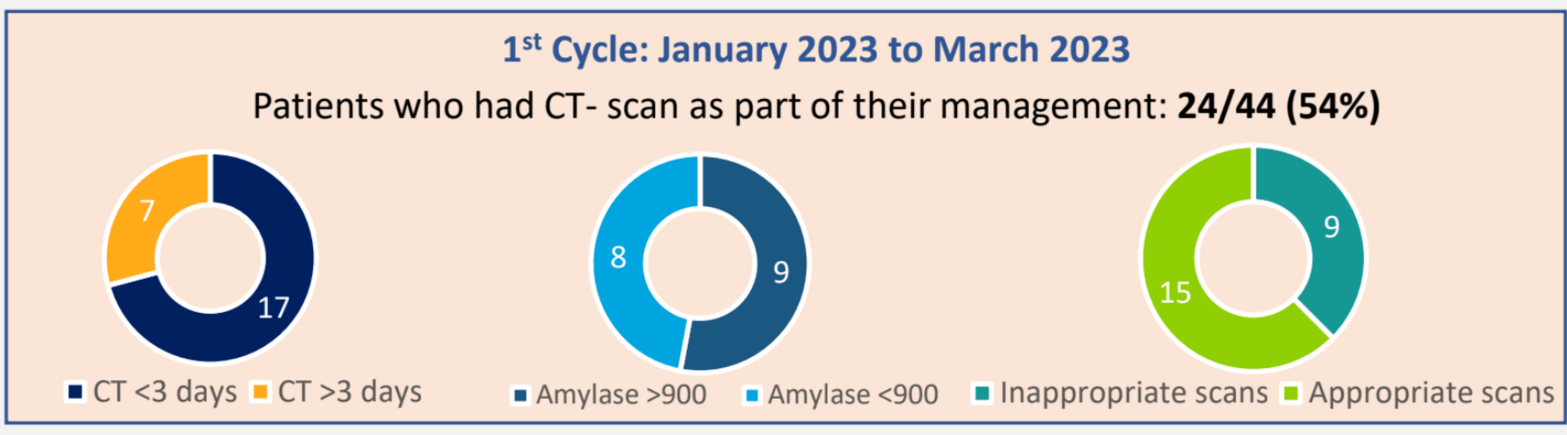
Results of the first cycle of the audit CT: computed tomography

In the first cycle, 44 patients were noted to have presented to the emergency department with symptoms suggestive of acute pancreatitis. Out of these patients, 54% (24) had a CT scan as a part of their management, of which 62.5% (15) were in less than 72 hours. Serum amylase was checked in all patients in the first cycle (Figure 1).

**Figure 2:**
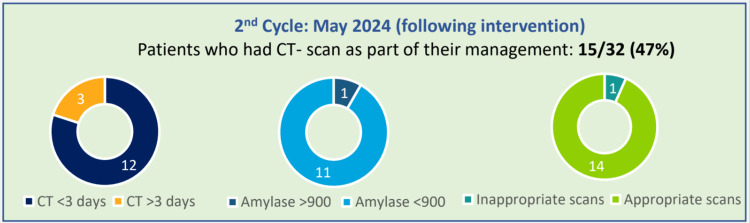
Results of the second cycle of the audit after intervention CT: computed tomography

In the second cycle, 32 patients were noted to have presented to the emergency department with symptoms suggestive of acute pancreatitis. Out of these patients, 46.8% (15) had a CT scan as a part of their management, of which 20% (3) had one within less than 72 hours. Serum amylase was checked in all patients in the second cycle (Figure 2).

Imaging utilization

Figure 1 shows the results from the first cycle of the audit: 54% of the patients had CT scans as part of their management, of which 62.5% (15) were within 72 hours of presenting with symptoms of suspected acute pancreatitis to the emergency department.

Figure 2 shows the results from the second cycle of the audit after the intervention. It showed 46.8% (15) of the patients had CT scans as part of their management, of which 20% (3) were within 72 hours of presenting with symptoms of suspected acute pancreatitis to the emergency department, thus showing improvement. It also showed that after the intervention, the number of inappropriate scans, the scans that were not in adherence with the standards, had reduced.

Figures 1-2 also show the proportion of patients having their serum amylase levels within normal limits and those who had their serum amylase levels elevated more than 900 U/L.

In the radiology request forms submitted by the clinicians, some common reasons included suspected necrosis, perforation, and bowel obstruction. Some patients were also mentioned to have had prior diagnoses of pancreatitis or gallstone disease.

Intervention outcomes

The data from the second cycle after the intervention showed reduced use of early CT scans and increased adherence to the standard recommendations.

In the re-audit following the intervention, only one patient (3%) had a CT scan (less than 72 hours from symptom onset) when the amylase was diagnostic. All other scans were requested as per guidance, such as due to diagnostic uncertainty, or after 72 hours to check for complications. This shows an improvement from the first audit cycle (21.4% early CT with diagnostic amylase). The p-value was found to be 0.0362 (Fisher's exact test). However, the limitation of statistical testing is that it is based on a small sample size. There was no change in the inclusion of pancreatitis severity within the CT reports.

## Discussion

This audit reveals important gaps in adherence to RCR imaging standards for acute pancreatitis within a district general hospital setting. The frequent use of early CT scans in Cycle 1, performed within 72 hours of presentation in over half of patients, is inconsistent with recommendations and exposes patients to unnecessary ionizing radiation without clear clinical benefit. This finding aligns with other published studies reporting overuse of early CT in acute pancreatitis [12,13]. A survey of radiologists and emergency physicians found that CT overuse in emergency departments is largely driven by diagnostic uncertainty, defensive medicine, and the involvement of less experienced staff. Additional contributors include limited time for clinical evaluation, inadequate medical education, and reliance on imaging to expedite patient flow [12].

Early CT scans have limited sensitivity for pancreatic necrosis, as inflammatory changes evolve over several days, potentially leading to underestimation of disease severity [13]. Overreliance on early imaging may lead to misinterpretation, unwarranted interventions, and inefficient resource use. In most patients with suspected acute pancreatitis, the diagnosis can be established clinically with characteristic pain and elevated enzymes, and CT performed within the first 48-72 hours rarely alters management. Early CT not only adds little diagnostic value but also risks underestimating necrosis and disease severity, which evolve more reliably after the 72-hour window [14]. In patients with uncomplicated acute pancreatitis, CT scans rarely reveal additional findings beyond what is established clinically and biochemically. Yet, their routine use led to nearly $950,000 in unnecessary costs in one cohort [15]. Avoiding these unwarranted scans reduces healthcare expenditure without affecting patient outcomes or hospital stay.

The underutilization of ultrasound within 24 hours, critical for identifying biliary causes of acute pancreatitis, represents a missed opportunity for timely diagnosis and management [3]. Ultrasound is widely accessible, noninvasive, and free from radiation exposure, making it ideal as a first-line imaging modality.

Interventions focusing on multidisciplinary education, departmental dissemination of audit findings, and visual reminders in clinical areas were effective in improving compliance in Cycle 2, reducing early CT usage, and increasing serum amylase testing. The intervention in this clinical audit placed strong emphasis on education to promote adherence to imaging guidelines. Findings from the first audit cycle were presented at departmental meetings, where discussions highlighted the importance of appropriate imaging and the potential harms of unnecessary CT use. To reinforce these messages, awareness posters were displayed in the A&E and General Surgery departments, serving as accessible visual reminders for staff in high-pressure clinical environments. In addition, targeted discussions with radiology staff helped to ensure the application of guidelines across departments.

This multifaceted educational approach proved effective, as it increased awareness of best practice, encouraged more thoughtful decision-making around imaging requests, and promoted collaboration between clinical teams. These outcomes align with previous studies demonstrating that audits combined with education and visual reminders can significantly reduce unwarranted imaging while supporting safe, evidence-based care [16].

While improvements were evident, there is still an opportunity to enhance further consistency in reporting timing and key prognostic CT features. Efforts to improve could focus on incorporating key prognostic features and scan timing into regular teaching sessions and further improving documentation quality, supporting more informed clinical decision-making.

Strengths and limitations

The audit benefits from a closed-loop design with a clear baseline assessment, targeted intervention, and reassessment. Limitations include a relatively small sample size, potential documentation inconsistencies, and the inability to capture all clinical decision-making nuances.

Implications and future directions

This study underscores the importance of adhering to imaging guidelines to reduce unnecessary radiation exposure, a public health priority considering cumulative ionizing radiation risks [4,5]. Ongoing education, multidisciplinary collaboration, and routine audits are essential to sustain improvements. Further research may explore barriers to guideline adherence, the impact of standardized reporting templates, and patient outcomes linked to imaging timing.

## Conclusions

This clinical audit at a district general hospital demonstrates that targeted quality improvement interventions can enhance adherence to imaging guidelines in acute pancreatitis. Reducing inappropriate early CT scans lowers radiation risks and optimizes resource use, while increased early ultrasound supports timely diagnosis of biliary pancreatitis. Sustained efforts and continuous audit cycles are recommended to embed best practices.
